# p66 Shc and tyrosine-phosphorylated Shc in primary breast tumors identify patients likely to relapse despite tamoxifen therapy

**DOI:** 10.1186/bcr1631

**Published:** 2006-12-29

**Authors:** A Raymond Frackelton, Li Lu, Pamela A Davol, Robert Bagdasaryan, Laurie J Hafer, Dennis C Sgroi

**Affiliations:** 1 Department of Research, Roger Williams Medical Center, 825 Chalkstone Avenue, Providence, RI 02908, USA; 2 Departments of Medicine and of Pathology and Laboratory Medicine, Brown University, Box G-RWH, Providence, RI 02912, USA; 3 Department of Surgery, Roger Williams Medical Center, 825 Chalkstone Avenue, Providence, RI 02908, USA; 4 Department of Pathology, Roger Williams Medical Center, 825 Chalkstone Avenue, Providence, RI 02908, USA; 5 Catalyst Oncology, Inc., One Innovation Drive, Worcester, MA 01605, USA; 6 Molecular Pathology Unit and Center for Cancer Research, Massachusetts General Hospital, Building 149, 13th Street, Charlestown, MA 02129, USA;Mailstop: Molecular Pathology Unit – 7149

## Abstract

**Introduction:**

Shc adapter proteins are secondary messenger proteins involved in various cellular pathways, including those mediating receptor tyrosine kinase signaling and apoptosis in response to stress. We have previously reported that high levels of tyrosine-phosphorylated Shc (PY-Shc) and low levels of its inhibitory p66 Shc isoform are strongly prognostic for identifying both early node-negative and more advanced, node-positive, primary breast cancers with high risk for recurrence. Because aberrant activation of tyrosine kinases upstream of Shc signaling proteins has been implicated in resistance to tamoxifen – the most widely prescribed drug for treatment of estrogen receptor-positive breast cancer – we hypothesized that Shc isoforms may identify patients at increased risk of relapsing despite tamoxifen treatment.

**Methods:**

Immunohistochemical analyses of PY-Shc and p66 Shc were performed on archival primary breast cancer tumors from a population-based cohort (60 patients, 9 relapses) and, for validation, an independent external cohort (31 patients, 13 relapses) in which all patients received tamoxifen as a sole systemic adjuvant prior to relapse.

**Results:**

By univariate and multivariate analyses, the Shc proteins were very strong and independent predictors of treatment failure in both the population-based cohort (interquartile hazard ratio = 8.3, 95% confidence interval [CI] 1.8 to 38, *P *= 0.007) and the validating cohort (interquartile relative risk = 12.1, 95% CI 1.7 to 86, *P *= 0.013).

**Conclusion:**

These results suggest that the levels of PY-Shc and p66 Shc proteins in primary tumors identify patients at high risk for relapsing despite treatment with tamoxifen and therefore with further validation may be useful in guiding clinicians to select alternative adjuvant treatment strategies.

## Introduction

Tamoxifen, a partial estrogen receptor (ER) antagonist and the most widely prescribed drug for the treatment of ER-positive breast cancer, provides a 40% to 50% decrease in the rate of relapse in this group of patients [1]. The precise mechanism of tamoxifen action is unknown; however, binding of tamoxifen to the ER inhibits estradiol activation of the receptor and ER-dependent proliferation and promotes cellular apoptosis [2,3]. Despite the objective responses or disease stabilization achieved with tamoxifen treatment in more than 50% of patients with previously untreated metastatic breast cancer [4], nearly 100% of metastatic patients and up to 40% of patients receiving adjuvant tamoxifen will relapse and die [1]. Clearly, there is a need to identify those patients who are at increased risk of relapse even though tamoxifen therapy has been administered: such patients would be candidates for chemotherapy or alternative adjuvant therapies such as Herceptin^® ^(Genentech, Inc., South San Francisco, CA, USA) and tyrosine kinase inhibitors.

Accumulating evidence indicates that active tyrosine kinases play a major role in innate and acquired tamoxifen resistance [3,5]. Ligand-bound ER has the ability to activate growth factor receptors and their downstream kinases through either direct cellular membrane interactions, as occur with HER2 and insulin-like growth factor receptor, or via downstream phosphorylation and transactivation events, as occur with the epidermal growth factor receptor [6,7]. Conversely, crosstalk with these receptors enhances the sensitivity of the ER to ligand and may lead to ligand-independent activation [8] or may hypersensitize ER to the normally weak estrogenic effects of tamoxifen [9].

Three Shc adapter proteins (p46, p52, and p66 Shc) are tyrosine-phosphorylated (PY) in response to signaling from ER as well as from these other growth factor receptors [10-12]. The PY-Shc proteins play key roles in activating the Grb2-SOS to Ras to mitogen-activated protein kinase pathway [13-15] and therefore the levels of PY-Shc might correspond to the likelihood of tamoxifen resistance. In contrast to the other Shc isoforms, however, p66 Shc functions as both a feedback downregulator of growth factor signaling [16,17] and as a critical facilitator of stress-induced apoptosis [18]. Thus, decreased tumor expression of p66 Shc may correspond to both increased likelihood of growth factor-mediated tamoxifen resistance and resistance to tamoxifen-driven apoptosis.

We hypothesized, therefore, that high levels of PY-Shc and low levels of p66 Shc in primary tumors may identify patients with increased risk of relapse despite having received tamoxifen therapy. Here, we report an affirmative test of this hypothesis in a population-based cohort and an independent, external validation cohort of patients, all of whom received first-line tamoxifen therapy as a sole systemic adjuvant.

## Materials and methods

### Patient populations

#### Population-based cohort

Formalin-fixed, primary tumors from 60 patients (9 relapses) with invasive breast cancer diagnosed between 1985 and 1991 were obtained from the archives of the Department of Pathology, Roger Williams Medical Center (Providence, RI, USA). Patients had surgical removal of the primary lesion (followed by local radiation if breast-conserving surgery was employed), and all patients received tamoxifen as the sole systemic adjuvant therapy until time of censorship or disease recurrence. Of the 60 patients, none was known to be ER-negative and 55 were ER-positive. ER positivity was defined as a dextran-coated charcoal ligand-binding assay (LBA) result of more than 10 fmol/mg protein [19]. Non-relapsing patients had a mean follow-up of 6.1 years, and relapsing patients had a mean time to first relapse of 3.4 years (0.08 to 7.4 years).

#### Validation cohort

Archival, formalin-fixed, paraffin-embedded primary tumors from 31 patients (13 relapses) were obtained as a subset of the case-control study by Ma *et al*. [20]. All patients received tamoxifen as part of their first course of therapy. No patients received neoadjuvant chemotherapy or other systemic adjuvant therapies before disease recurrence. All patients were ER-positive, defined as more than 10 fmol/mg protein by dextran-coated charcoal LBA or as an immunohistochemical result of more than 10% nuclear staining with a score of more than 3 using a monoclonal ER antibody, clone 1D5 (Dako North America, Inc., Carpinteria, CA, USA). To minimize confounding of potential predictive markers by clinicopathological characteristics, relapsing and non-relapsing patients were closely matched by ER/progesterone receptor (PR) status, nodal status, tumor grade, patient age at diagnosis, and tumor size. Non-relapsing patients had a mean follow-up of 8.7 years, and relapsing patients had a mean time to first recurrence of 5.3 years (0.5 to 11.2 years).

### Antibodies and immunohistochemical analysis

Characterization of the affinity-purified antibodies to PY-Shc and p66 Shc and the immunohistochemical procedures have been described previously [21]. An average staining intensity for all invasive cancer cells in a section was determined [22,23]: staining intensities of cancer cells were scored on a scale of 0 to 5 [21] by a scorer blinded to patient outcomes and clinicopathological characteristics. To accurately reflect the heterogeneity in the tumor staining, the intensity score was multiplied by the percentage of invasive cancer cells staining at each intensity. These results were summed and then divided by 100 to achieve a scale of 0 (no invasive cancer cells stained) to 5 (all invasive cancer cells stained maximally). From the population-based study, a random subset of slides (*n *= 24) was scored independently by a second scorer blinded both to patient outcomes and to the other scorer's results. A concordance of 0.96 between the scorers, with regard to the assignment of patients to high versus low ratios of PY-Shc to p66 Shc staining, was observed, with a Pearson correlation coefficient of 0.80 (*P *< 0.0001) [21].

### Statistical considerations

Statistical calculations were performed using the Stata statistical package (Stata version 8.0; StataCorp LP, College Station, TX, USA). Clinicopathological characteristics were analyzed by univariate log-rank analysis of their Kaplan-Meier survivor functions. Differences between PY-Shc-to-p66 Shc ratios in relapsing and non-relapsing patients were tested for significance by Wilcoxon rank sum test. The ability of PY-Shc and p66 Shc, as continuous variables, to predict disease recurrence was analyzed in univariate and multivariate Cox proportional hazards models with relapse-free survival (RFS) as an endpoint. RFS was defined as the time from surgical removal of the tumor until first clinical recurrence or loss to follow-up (censored). Cox models were checked for validity of proportional hazards assumptions and goodness of fit using Schoenfeld and Cox-Snell residual analyses, respectively. Covariate improvement to models was tested for significance by partial likelihood ratio (pLR) analysis. For visual evaluation of RFS, Kaplan-Meier survival functions were constructed using PY-Shc and p66 Shc values dichotomized on their median values, and differences between the dichotomized Shc variables were tested by univariate log-rank analysis. All statistical tests were two-sided, with significance taken as *P *< 0.05.

## Results

In the population-based cohort, univariate log-rank analysis of RFS identified significant predictive values for nodal status and tumor stage and values approaching significance for tumor grade and PR status. In the validation cohort, however, no clinicopathological characteristic attained predictive significance (Table 1). This was expected inasmuch as the validation cohort was a subset of patients from a case-control study in which relapsing and non-relapsing patients had been closely matched by ER/PR status, nodal status, and tumor size [20]. The case-control validation cohort, therefore, provided a means to evaluate the Shc predictive markers with minimal potential confounding by clinicopathological variables.

**Figure 1:**
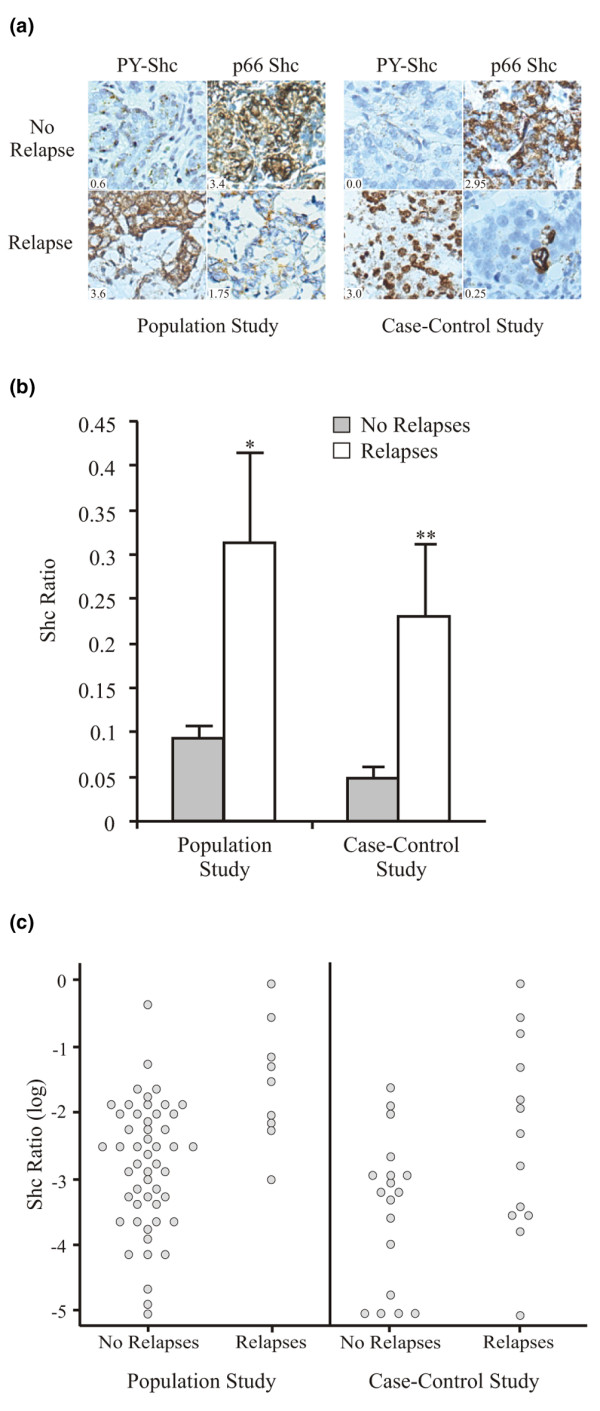
Comparison of the PY-Shc-to-p66 Shc ratio in primary tumors as a function of disease recurrence. **(a) **Photomicrographs of PY-Shc and p66 Shc immunohistochemical staining patterns in primary tumors from patients who received tamoxifen and then either relapsed or did not relapse. Shc scores are shown in the insets. The left panel shows the staining patterns of two patients from the population-based cohort, and the right panel shows the staining patterns of two patients from the case-control cohort. Total magnification, ×200. **(b) **The ratio of PY-Shc to p66 Shc (Shc ratio), scaled 0 to 1, is shown (mean ± standard error) for primary tumors from patients who either relapsed or did not relapse. A comparison of the mean Shc ratios for patients in the population-based study with or without relapses is shown on the left side of the column graph (50 no relapses, 9 relapses; *P = 0.002). A comparison of the mean Shc ratios for patients in the case-control study with or without relapses is shown on the right side of the column graph (18 no relapses, 13 relapses; **P = 0.049). **(c) **Scatter histograms of the Shc ratio, on a log scale, observed in the patients' primary tumors as a function of relapse in the population-based and case-control cohorts. PY-Shc, tyrosine-phosphorylated Shc.

Results representative of the immunohistochemical analyses of primary tumors shown in Figure 1a depict the contrast typically observed between PY-Shc and p66 Shc staining intensities in cancer cells from patients who relapsed despite therapy compared to those who did not. Based on a previously established scoring system for Shc staining intensity [21], patients in both studies who relapsed (irrespective of tamoxifen therapy) had primary tumors with a higher PY-Shc-to-p66 Shc staining ratio compared to those tumors from patients who did not relapse (0.31 ± 0.10 versus 0.09 ± 0.02, *P *= 0.002, and 0.23 ± 0.08 versus 0.05 ± 0.01, *P *= 0.049, in the population-based and validation cohorts, respectively; Figure 1b,c).

**Figure 2:**
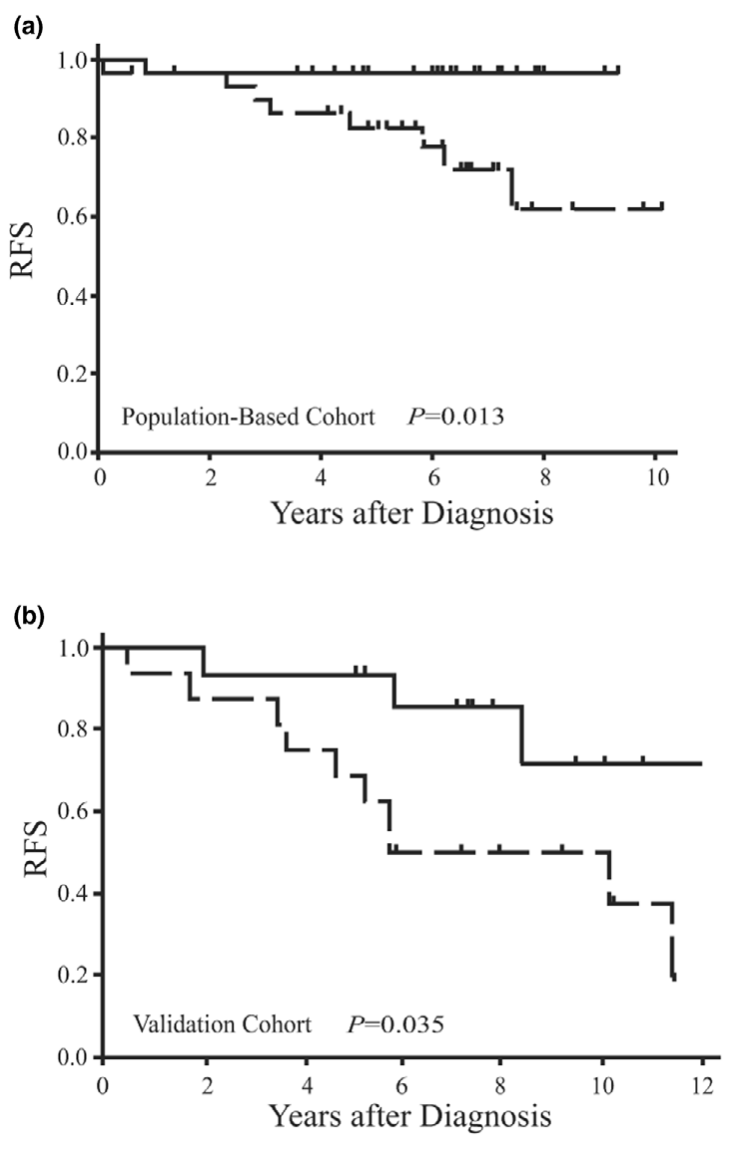
Relapse-free survival (RFS) according to Shc in patients with breast cancer. **(a) **Kaplan-Meier graphical analysis of RFS in patients from the population-based cohort partitioned by the median value for Shc (*P *= 0.013). **(b) **Kaplan-Meier graphical analysis of RFS in patients from the case-control cohort partitioned by the median value for Shc (*P *= 0.035).

Consistent with these observations, by univariate Cox proportional hazards analysis, significant increases in risk of relapse in these tamoxifen-treated patients were observed with increasing values of the PY-Shc-to-p66 Shc ratio in each cohort. The increase in risk with PY-Shc-to-p66 Shc ratio can be appreciated by the large hazard ratio (HR) for the full observed range of patient scores: 14 (95% confidence interval [CI]) 2.1 to 93, *P *= 0.006) for the population-based cohort and 43 (95% CI 4 to 480, *P *= 0.002) for the validation cohort. Considering the PY-Shc and p66 Shc variables as mutually adjusted covariates, rather than constrained as the ratio, improves model fit considerably [21]. Compared to the ratio in the present study, PY-Shc and p66 Shc considered together as covariates improved model fit (by pLR analysis for the significance of the change in chi-squared [Δχ^2^] for n degrees of freedom [Δdf]: pLR Δχ^2 ^= 6.08, Δdf = 1, *P *= 0.013) and provided an HR for the full observed range of Shc values of 240 (95% CI 9.8 to 5,900, *P *= 0.001) and an interquartile HR of 8.6 in the population-based cohort (Table 2). For the validation cohort, a full-range HR of 53 (95% CI 1.8 to 1,600, *P *= 0.02) and an interquartile HR of 7.7 were observed (Table 2). This relationship between the Shc scores and relapse in tamoxifen-treated patients in both studies is easily visualized from the Kaplan-Meier RFS functions. The median values of the composite PY-Shc and p66 Shc scores from each cohort were used as a cut point to divide the patients into high- and low-risk groups. Within 8 years of primary diagnosis, 38% and 50% of high-risk tamoxifen-treated patients in the population-based and validation cohorts, respectively, relapsed, whereas only 3.3% and 14% of the low-risk patients, respectively, relapsed (Figure 2).

By multivariate analysis, the ability of the Shc proteins to predict relapse despite tamoxifen therapy was independent of nodal status, AJCC (American Joint Committee on Cancer) stage (population-based cohort), tumor stage, tumor grade, PR status, and patient age at diagnosis. Of these clinical variables, only nodal status remained as a significant covariate (population-based cohort: *P *= 0.04; validation cohort: *P *= 0.04), with the Shc proteins retaining their full prognostic value and strength (Table 2). Inclusion of nodal status improved the overall fit of the model, suggesting that it may be useful as an independent predictive covariate along with PY-Shc and p66 Shc.

## Discussion

In women with ER-positive breast tumors, the high initial response rate to tamoxifen has made this drug the most widely prescribed treatment for this group of patients. Aberrant activation of kinase signaling pathways in invasive breast cancer cells, however, may play a widespread role in failure of tamoxifen therapy [3,5]. Such pathways have the potential to bypass ER-dependent growth of cells or recruit ERs independent of ligand activation through crosstalk, thus facilitating a resistant phenotype while confounding the predictive utility of standard clinical markers. Therefore, it is not surprising that many investigators have focused on identifying new molecular markers that may identify patients with high risk of relapse despite tamoxifen therapy. Supporting such an approach, the application of a multimarker predictive model evaluating the interdependency between ER, PR, HER2/neu, bcl-2, myc, and tp53 has resulted in superior predictive power for failure of tamoxifen therapy, compared to standard marker evaluations alone [22].

In another approach, the expression levels of large numbers of genes have been used to predict disease outcome [23]. Despite the promising results obtained from these microarray gene analyses-based studies, increased costs and complex methodology may limit their widespread application. Additionally, gene-expression analysis does not measure the actual level of protein expression, post-translational modification, or aberrant subcellular localization in the invasive cancer cells, potentially limiting this methodology's accuracy for predicting individual patient risk.

In this study, we analyzed the expression and post-translational modification of Shc adapter proteins in two independent cohorts of patients with breast cancer by a semi-quantitative immunohistochemistry (IHC) staining method and found the Shc proteins to be strongly predictive for disease recurrence in tamoxifen-treated patients. Primary tumors of these patients who subsequently relapsed contained characteristically higher levels of PY-Shc, lower levels of p66 Shc protein, or both compared to primary tumors of those patients who did not relapse. In Cox survival models, the levels of PY-Shc and p66 Shc could be modeled as the ratio of PY-Shc to p66 Shc or (more effectively) as linked continuous covariates, showing a very strong ability to predict patient outcome (Table 2). These observations support the hypothesized molecular model of tamoxifen resistance, in which PY-Shc and p66 Shc subserve at least partially opposing molecular functions.

Although the predictive ability of markers is most robust when their expression can be considered as continuous variables [24], categorizing the Shc scores into groups with high and low risk of relapse despite tamoxifen therapy is useful for comparing RFS curves (Figure 2). By univariate log-rank analysis, RFS for high- and low-risk groups in each cohort was significantly different. Tamoxifen-treated patients with tumors having a high PY-Shc and/or low p66 Shc score experienced a significantly shorter RFS compared to tamoxifen-treated patients with tumors having a low PY-Shc and/or high p66 Shc score.

Discussions of multimarker prediction models often present HRs or relative risks (RRs) that represent the full range of risk over which the model can assign patients. A clinically more informative measure, however, is the interquartile RR: the range of risk that can be observed within the middle 50% of patients. The Shc proteins assigned a very wide range of risk of relapse over the middle 50% of patients, with an interquartile RR of 8 (Table 2) in the population-based cohort and 12 (Table 2) in the validation cohort. The validation cohort was a subset of the case-controlled patients studied by Ma *et al*. [20], and its Shc interquartile RR of 12 is comparable to the RR of 7 reported by Ma *et al*. [20] for the expression ratio of HOXB13/IL-17BR receptor in a multivariate model for these same patients.

In multivariate analysis, the ability of the Shc proteins to predict relapse in tamoxifen-treated patients was strong and independent of all potential covariates. Of these, only nodal status remained as a marginally significant covariate in each cohort, with the Shc proteins retaining their full prognostic value and strength (Table 2). Inclusion of nodal status improved the overall fit of the model, suggesting that it may be useful as a predictive covariate along with PY-Shc and p66 Shc. Consistent with our previous report [21] of the Shc proteins predicting outcome for both node-negative and node-positive patients, neither by analysis of correlation nor interaction in the Cox model was there evidence of association between the Shc proteins and nodal status. The independence of nodal status as a predictor is also consistent with the findings of a large meta-analysis in which node-positive compared to node-negative patients were shown to have approximately twice the absolute risk of failing tamoxifen therapy [1].

Other potential covariates may attain significance when larger study populations are examined. For example, to the extent that HER2/neu overexpression and activation [3,25] correlate with both tamoxifen resistance and its activation signals through Shc-associated pathways, the predictive value of Shc may occur, in part, due to its capacity as a downstream reporter of HER2/neu activity. However, HER2/neu is activated in only approximately 16% of ER-positive patients [26]. For the majority of cases of tamoxifen resistance, the specific etiology remains unknown, although there is some evidence that tamoxifen-induced apoptosis may play a role [27]. Additionally, in other studies, we have observed that the ability of Shc proteins to predict patient outcome is independent of HER2/neu expression levels (unpublished data).

All of the patients' tumors in these studies expressed ER as determined by LBA. Tumors that are ER-positive by LBA constitute approximately 90% of breast tumors that are ER-positive, as judged by currently used, more sensitive IHC assays [28,29]. It will be important in subsequent large validation studies to determine whether the Shc proteins are similarly able to predict relapse after tamoxifen therapy for this small subgroup of patients whose tumors express low amounts of ER.

The difference in incidence of relapse in the population-based study (15%, 9/40) and the higher incidence in the validation study (42%, 13/31) reflects the fact that the validation cohort is a subset of the Ma *et al*. [20] case-control study, in which equal numbers of relapsing and non-relapsing patients were selected based on closely paired clinicopathological characteristics. This approach was implemented to minimize variable heterogeneity that could potentially confound the analysis of the predictive abilities of Shc proteins. The case-control design provided ample statistical power with a minimal total number of patients in which to validate our primary hypothesis generated from the population-based study: namely, that the Shc proteins identify patients at high risk of relapse despite tamoxifen therapy. However, this selection procedure does not make possible an exact comparison of the magnitude of the predictive abilities of the Shc proteins (HR and RR) between the two studies. Despite this, the significant and very strong RR in the case-control cohort affirms the results of the population-based study showing the predictive independence of the Shc proteins from potentially confounding clinicopathological characteristics.

## Conclusion

The very strong and independent ability of the Shc proteins to predict disease relapse despite tamoxifen treatment reported here, coupled with their well-validated ability to predict relapse in untreated patients, provides a compelling argument for validating these results in larger patient cohorts and randomized trials capable of determining the ability of the Shc proteins to predict tamoxifen benefit. Furthermore, compared to gene array-based methodologies, the application of state-specific antibodies in a standardized immunohistochemical analysis provides a practical and cost-effective approach with the advantage of evaluating post-translational protein modifications in addition to expression levels. Upon further successful validation of these results, high PY-Shc and low p66 Shc expression levels in primary tumors should be clinically useful in predicting patients at high risk of relapse despite tamoxifen therapy and thus in identifying those patients who may benefit from adjuvant chemotherapy or targeted therapies such as Herceptin^® ^and tyrosine kinase inhibitors. Conversely, low PY-Shc and high p66 Shc expression levels in primary tumors may identify patients at low risk of relapse after tamoxifen therapy and thus spare them the costs and toxicities of additional treatments.

## Abbreviations

Δχ^2 ^= change in chi-squared; CI = confidence interval; df = degrees of freedom; ER = estrogen receptor; HR = hazard ratio; IHC = immunohistochemistry; LBA = ligand-binding assay; pLR = partial likelihood ratio; PR = progesterone receptor; PY = tyrosine-phosphorylated; RFS = relapse-free survival; RR = relative risk.

## Competing interests

ARF is an employee of Catalyst Oncology, Inc. (Worcester, MA, USA) and a co-inventor on a patent of the IHC assay for Shc. PAD is a co-inventor on a patent of the IHC assay for Shc. LJH is an employee of Catalyst Oncology, Inc. The remaining authors declare that they have no competing interests.

## Authors' contributions

ARF conceived the study, participated in its design and execution and in the drafting of the manuscript, and conducted the statistical analyses. LL, PAD, and LJH participated in IHC staining and analysis and in the drafting of the manuscript. RB had an integral role in the analysis of the IHC staining. DCS participated in the study design and in the acquisition and production of the case-control specimens. All authors read and approved the final manuscript.

## Figures and Tables

**Table 1:** Clinicopathological characteristics and univariate analysis of ability to predict failure of tamoxifen therapy

	Population-based cohort				Case-control cohort			
			RFS				RFS	

Variable	*n*^a^	Percentage^b^	8 years	*P*^c^	*n*^a^	Percentage^b^	8 years	*P*^c^

All patients	9/60	15	79		13/31	42	67	
Nodal status	9/60	15		0.04	13/28	46		0.30
Negative	4/45	9	85		8/15	53	57	
Positive	5/15	33	64		5/13	38	71	
AJCC stage	9/60	15		0.16	ND^d^	ND^d^		
1	2/28	7	92					
2a	0/3	0	100^e^					
2b	7/27	26	58					
3a	0/1	0	100^e^					
Tumor stage	9/60	15		0.01	13/31	42		0.17
1	2/32	6	93		3/12	25	74	
2	4/21	21	55		10/19	53	62	
3	3/4	75	50^e^					
4	0/1	0	100					
Tumor grade	9/60	15		0.06	13/31	42		0.56
1	0/5	0	100		0/1	0	100	
2	2/22	9	82		8/19	45	63	
3	7/26	27	69		5/11	40	70	
PR status	9/60	15		0.06	13/31	47		0.96
Negative	0/16	0	100		3/6	50	67	
Positive	6/36	17	71		10/25	40	67	
Age	9/60	15		0.15	13/31	42		0.56
<50 years	2/6	33	83^e^		2/2	100	100	
≥50 years	7/54	13	84		11/29	38	64	

^a^9/60 indicates that there were 9 treatment failures out of 60 patients with known values for the indicated clinical characteristic. ^b^The percentage of treatment failures for the indicated clinical characteristic. ^c^*P *is given for log-rank (or log-rank trend) univariate analysis. Sensitivity tests conducted with missing values provided no evidence for selection bias. ^d^Clinical characteristic was not determined. ^e^For patients with AJCC stage 2a and 3a disease and patients less than 50 years old, the RFS is reported at 7 years. For patients with tumor stage 3 disease, the RFS is reported at 6 years. AJCC, American Joint Committee on Cancer; PR, progesterone receptor; RFS, relapse-free survival.

**Table 2:** Multivariate analyses of ability of Shc proteins to predict failure of tamoxifen therapy

	Population cohort		Case-control cohort	
Model	HR^a ^(95% CI)	*P*^b^	RR^a ^(95% CI)	*P*^b^
PY-Shc, p66 Shc	8.6 (2.6–30)	0.001	7.7 (1.4–44)	0.022
PY-Shc, p66 Shc				
Adjusted for nodal status	8.3 (1.8–38)	0.007	12.1 (1.7–86)	0.013

^a^HR (interquartile) is the RR of relapse comparing patients in the 75th percentile of PY-Shc and 25th percentile of p66 Shc to patients in the 25th percentile of PY-Shc and 75th percentile of p66 Shc. The multivariate Cox model containing PY-Shc and p66 Shc was adjusted for nodal status, AJCC (American Joint Committee on Cancer) stage (population-based cohort), tumor stage, tumor grade, progesterone receptor status, and patient age at diagnosis. ^b^Significance using Wald statistic. CI, confidence interval; HR, hazard ratio; PY-Shc, tyrosine-phosphorylated Shc; RR, relative risk.
